# Impact of family history of alcoholism on glutamine/glutamate ratio in anterior cingulate cortex in substance-naïve adolescents

**DOI:** 10.1016/j.dcn.2015.04.005

**Published:** 2015-04-23

**Authors:** Julia E. Cohen-Gilbert, Jennifer T. Sneider, David J. Crowley, Isabelle M. Rosso, J. Eric Jensen, Marisa M. Silveri

**Affiliations:** a McLean Imaging Center, McLean Hospital, 115 Mill Street, Mail Stop 204, Belmont, MA 02478, USA; b Department of Psychiatry, Harvard Medical School, 401 Park Drive, 2-West, Boston, MA 02215, USA

**Keywords:** Alcoholism, Family history, MRS, Adolescence, Glutamate, Impulsivity

## Abstract

Neuroimaging studies of individuals with family histories of alcoholism provide evidence suggesting neurobiological risk factors for alcoholism. Youth family history positive (FH+) for alcoholism exhibit increased impulsivity compared to family history negative (FH−) peers in conjunction with altered functional activation in prefrontal cortex, including anterior cingulate cortex (ACC). This study examined glutamate (Glu) and glutamine (Gln), amino acids vital to protein synthesis, cellular metabolism and neurotransmission, acquired from ACC and parieto-occipital cortex (POC) using magnetic resonance spectroscopy (MRS) at 4T. Participants were 28 adolescents (13 male, 12–14 yrs) and 31 emerging adults (16 male, 18–25 yrs), stratified into FH− and FH+ groups. Significantly higher ACC Gln/Glu was observed in emerging adults versus adolescents in FH− but not FH+ groups. In FH− adolescents, higher impulsivity was significantly associated with higher ACC Gln/Glu. In FH+ emerging adults, higher impulsivity was negatively associated with ACC Gln/Glu. No differences or associations were observed for POC. These findings provide preliminary evidence that family history of alcoholism is associated with a neurochemical profile that may influence normative age differences in glutamatergic metabolites and their association with impulse control, which together could confer greater genetic risk of addiction later in life.

## Highlights

• Family history of alcoholism was studied with MRS in adolescents and emerging adults.
• Glutamine/glutamate ratio was measured at 4T to index glutamate neurotransmission.
• Within FH−, emerging adults had significantly higher Gln/Glu ratios than adolescents.
• Within FH+, no age-related differences were observed in Gln/Glu ratios.
• Gln/Glu correlated with impulsivity in FH− adolescents and FH+ emerging adults.

## 1 Introduction

Alcohol use typically begins during adolescence, a time during which the still-maturing brain may be particularly vulnerable to its neurotoxic effects. However, while a number of differences in brain structure and function associated with early alcohol drinking have been identified (Squeglia et al., 2009), it remains unclear whether such neurological changes are antecedent or consequent to adolescent alcohol use. A family history of alcoholism (FH) constitutes a major risk factor for the development of alcohol use disorders (Conway et al., 2003). Thus, neuroimaging studies of adolescents with family histories of alcoholism can help identify neurobiological risk factors for alcohol use disorders and can help distinguish potential precursors of alcoholism from its sequelae. A number of neurobiological and behavioral differences have been identified between youth who are family history positive (FH+) for alcoholism and family history negative (FH−) peers, including increased impulsivity (Gierski et al., 2013) and differences in recruitment of regulatory brain regions during inhibitory demands, as revealed by functional magnetic resonance imaging (fMRI) (DeVito et al., 2013, Hardee et al., 2014, Silveri et al., 2011). While the role of neurochemistry in older adults with alcohol use disorders has been investigated (Meyerhoff et al., 2013, Meyerhoff et al., 2014, Sullivan and Pfefferbaum, 2014), neurochemical correlates of family history of alcoholism in youth has remained largely unstudied to date. To this end, the current study employed magnetic resonance spectroscopy (MRS) to investigate the glutamatergic system, including turnover of the general metabolic pool, as indexed by the ratio of glutamine (Gln) to glutamate (Glu) (Öngür et al., 2008, Yüksel and Öngür, 2010) in FH− and FH+ adolescents and emerging adults. Relationships between Gln/Glu and impulsivity were also examined in these populations.

Maturation of frontal brain regions, including the anterior cingulate cortex (ACC), supports normative reductions in impulsivity during adolescence, as regulatory brain circuitry follows a protracted developmental course that extends well into early adulthood (Casey et al., 2008, Gogtay et al., 2004, Luna and Sweeney, 2004, Rubia et al., 2006). Difficulty in regulation of impulsive actions during this age period may contribute to experimentation with alcohol, drinking binges, and alcohol abuse in youth. Furthermore, inhibitory control is prominent among the cognitive abilities most vulnerable to disruption by alcohol (Sher et al., 1997), leaving adolescents who drink alcohol at an enhanced risk for reckless decision-making and continued substance misuse. Moreover, age of initiation, magnitude of use, and prevalence of alcohol use disorders are influenced by genetic vulnerability, as early onset and greater use and abuse have been linked with having a positive family history of alcoholism among adolescents and young adults (Biederman et al., 2000, Conway et al., 2003, Hill et al., 2000). Although intellectual functioning in this at-risk population typically falls within the healthy range (Alterman et al., 1989), children of alcoholics demonstrate deficits in abstract reasoning and planning, lower IQ scores, and poorer spelling and math performance compared to children of non-alcoholics (Poon et al., 2000). Further, altered executive functioning has been reported in FH+ youth in conjunction with increased self-report ratings of impulsivity (Gierski et al., 2013) and fMRI studies have identified numerous brain areas that are differentially recruited by FH+ and FH− individuals during tasks requiring cognitive control. For instance, studies have reported increased ACC activation in FH+ versus FH− participants during inhibitory control on classic tasks such as the Stroop Color–Word Interference task (Silveri et al., 2011) and the Go–NoGo task (Hardee et al., 2014, Jamadar et al., 2012). Collectively, prior data suggest that differential ACC functioning may have an influential role in elevating risk for substance use and abuse in FH+ compared to FH− youth.

In recent years, high magnetic field strengths and advances in magnetic resonance spectroscopy (MRS) acquisition sequences have permitted investigation of important in vivo neurochemicals implicated in alcohol action, such as glutamate and gamma amino butyric acid (GABA) (Meyerhoff et al., 2014, Silveri, 2014). Notably, glutamate (Glu) and glutamine (Gln) can be resolved and quantified separately, particularly at high field (Hu et al., 2007, Jensen et al., 2009), allowing investigation of these amino acids that are vital to protein construction, cellular metabolism, and excitatory neurotransmission (Behar and Rothman, 2001, Patel et al., 2005, Sibson et al., 1998, Yüksel and Öngür, 2010). The ratio of Gln/Glu, thought to reflect Gln-Glu cycling, has been established as a potential index of neurotransmission (Yüksel and Öngür, 2010). Levels of Gln and Glu are of particular interest in alcohol research as alcohol use, dependence, and withdrawal have been shown to be associated with both acute and protracted alterations in glutamatergic systems (Hermann et al., 2012, Krystal et al., 2003, Tsai and Coyle, 1998). To date, however, only one study has used MRS to identify neurochemical correlates of risk for substance abuse in a FH+ population (Moss et al., 1997), in which phosphorous (^31^P) MRS was applied to study peripubertal FH+ adolescents and a low-risk FH− comparison group, demonstrating lower parietal phosphodiester concentrations in the high-risk adolescents who also presented with behavior disorders.

Accordingly, the current study is the first to use proton (^1^H) MRS to examine the association of age and FH status with markers of glutamatergic neurotransmission in ACC and a control region in parieto-occipital cortex (POC). It also evaluates relationships between ACC Glu/Gln ratios and cognitive and self-report measures of impulsivity in adolescents and emerging adults. It was hypothesized that the FH+ group would exhibit higher Gln/Glu ratios in ACC, but not in POC, compared to FH− counterparts, given increased vulnerability and relevance of the frontal lobe to addiction. Further, higher ACC Gln/Glu ratios were hypothesized to correlate positively with higher behavioral and self-report measures of impulsivity. Positive associations between Gln/Glu, FH+ status and impulsivity were predicted for both adolescent and emerging adult groups.

## 2 Materials and methods

### 2.1 Participants

Participants were 28 healthy adolescents (13 male), aged 12–14 years, and 31 healthy emerging adults (16 male), aged 18–25 years, stratified within each age group into low (FH−) or high (FH+) familial risk for alcoholism. Participants (all adolescents and a subset of emerging adults) were included in another published MRS study that investigated brain GABA levels using MEGAPRESS (Silveri et al., 2013). Participants were 96.7% non-Hispanic, 70.0% Caucasian. A summary of participant demographics is provided in Table 1. Participants reported no past or current psychological or neurological illnesses, history of head trauma, MRI contraindications, severe medical problems or psychoactive substance use. A urine screen was completed prior to scanning to rule out current psychoactive substance use and pregnancy. The Kiddie Schedule for Affective Disorder and Schizophrenia (KSADS) was used to rule out Axis I diagnoses in adolescents and the Structured Clinical Interview for DSM-IV Disorders (SCID) was used for emerging adults. Individuals were recruited from the community and were financially compensated for study participation. Adolescent subjects reported less than three episodes of lifetime alcohol use and no history of drug use. Emerging adults were non-smoking, light drinkers (alcohol use never exceeded three drinks per occasion, or five total occasions within a 30 day period for the duration of each individual's lifetime drinking history) who had minimal to no history of illicit substance use (less than 10 lifetime events). Parents/guardians of adolescent participants provided written informed consent and adolescent participants provided written assent prior to study participation. Emerging adults provided written informed consent prior to study participation. This study was approved by and conducted in accordance with the McLean Hospital/Partners Healthcare Internal Review Board.

**Table 1:** Participant demographics.

	Adolescent			Emerging adult		
	FH−	FH+	Total	FH−	FH+	Total
N	19	12	31	22	7	29
Female	12	4	16	12	2	14
Right handed	17	10	27	22	7	28
Age	13.6 (.84)	13.7 (.97)	13.6 (.88)	21.7 (1.6)	21.0 (1.9)	21.5 (1.7)
Education	7.21 (.98)	7.36 (.92)	7.27 (.94)	14.9 (1.5)	14.0 (1.5)	14.7 (1.5)
FH density	–	.42 (.27)	–	–	.57 (.31)	-

### 2.2 Assessment of family history of alcoholism

Emerging adult participants and accompanying parents of adolescent participants underwent a Family History–Epidemiologic (FHE) structured interview and an unstructured family interview to obtain information about substance use disorders in second-degree relatives. This information was used to stratify subjects into FH− or FH+ groups. Individuals in the FH+ group each had at least one biological parent or grandparent with a diagnosed alcohol use disorder. Of note, no participants reported having an alcohol-dependent mother, alleviating concerns over possible confounding effects of in utero alcohol exposure. Family expression of alcoholism, or family history density (FHD) of alcoholism, was calculated for FH+ participants (Table 1), with a single parent with a history of alcoholism contributing 0.5 and a single grandparent 0.25, for a range of 0.0 (FH−) to 2.0 (0.25–2.0, FH+) (Stoltenberg et al., 1998).

### 2.3 Measures of impulsiveness

All participants completed the Barratt Impulsiveness Scale (BIS-11) (Fossati et al., 2002) to assess trait impulsivity, including total score and attention (rapid shifts in attention/impatience with complexity), motor (impetuous action), and non-planning (lack of future orientation) subscale impulsivity scores. Two cognitive tasks were used to assess response inhibition: a modified Stroop test (time to complete and number of errors on Color Naming, Word Reading and Interference conditions) and a computerized Go–NoGo task (percent error rate on NoGo trials) (Finn et al., 1999, Leland et al., 2008; see also, Silveri et al., 2013).

### 2.4 Magnetic resonance imaging/spectroscopy

**Fig. 1:**
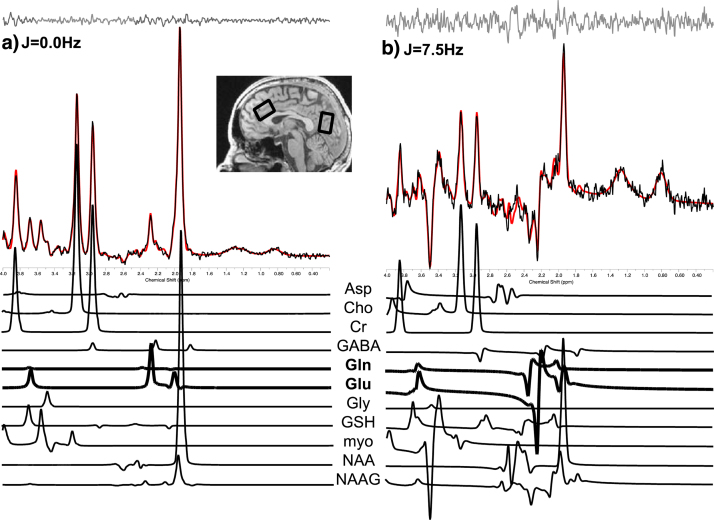
Sample ACC spectra acquired from a study participant (image inset, left voxel). Raw spectral (black) extractions from J = 0.0 Hz (a) and J = 7.5 Hz (b), out of the 64 J-resolved extractions used for fitting across the entire spectral surface (Jensen et al., 2009), are presented with accompanying LCModel fits (red). Stack plots of fitted metabolite components from 2D-JPRESS data sets are presented below each corresponding sample spectrum, highlighting Glu and Gln spectral peaks in bold for these two representative extractions. (For interpretation of the references to color in this figure legend, the reader is referred to the web version of the article.)

Magnetic resonance imaging (MRI) and proton (^1^H) MRS data were acquired at 4.0 Tesla (T) on a Varian Unity/INOVA whole-body MRI/MRS scanner (Varian Inc., Palo Alto, CA) and using a volumetric head coil (XLR Imaging, London, Canada). Head placement was confirmed for each participant using three-plane scout images. Following global shimming, high-contrast 3D fast low-angle shot T1-weighted images were acquired for placement of a 20 mm × 20 mm × 30 mm voxel in midline ACC and a 20 mm × 20 mm × 30 mm voxel in midline POC (Fig. 1, inset) (Silveri et al., 2013).

Manual voxel shimming yielded water linewidths that ranged from 9–12 Hz. Proton spectroscopy data were acquired using Point-Resolved Echo Spectroscopy Sequence modified for the current *J*-resolved ^1^H-MRS protocol (2D-JPRESS) to collect 24 TE-stepped spectra in each voxel region (Jensen et al., 2009). Each voxel was shimmed manually and was followed by water suppression. The 2D-JPRESS sequence collected 24 TE-stepped spectra (echo times from 30 to 490 ms in 20 ms increments), which provided sufficient *J*-resolved bandwidth (50 Hz) to resolve Glu from Gln. Additional acquisition parameters included TR = 2 s, f1 acquisition bandwidth = 50 Hz, spectral bandwidth = 2 kHz, readout duration = 512 ms, NEX = 16, total scan duration = 13 min. Unsuppressed water spectra were acquired immediately following metabolite acquisitions using the same 2D-JPRESS sequence to collect 24 TE-stepped water scans using 2D JPRESS parameters, but with 4 averages per TE-step.

All spectroscopic data processing and analyses were performed using in-house reconstruction code and LCModel fitting software (Provencher, 1993). In order to quantify proton metabolites (e.g., Glu and Gln), 24 TE-stepped free-induction decay series were zero-filled out to 64 points, Gaussian-filtered to minimize residual arising from NAA and Cr signals, and Fourier-transformed in the TE dimension. This resulted in 64 *J*-resolved spectra over 50 Hz. Using GAMMA-simulated *J*-resolved basis sets, every *J*-resolved spectral extraction (bandwidth of 50 Hz) was fit with its theoretically correct LCModel template (Smith et al., 1994). The integrated area under the entire 2D surface for each metabolite was calculated by summing raw peak areas across all 64 *J*-resolved extractions (for methodological details see Jensen et al., 2009). Sample ACC spectra (black) from the *J* = 0.0 Hz and *J* = 7.5 Hz extractions (out of the possible 64 *J*-resolved extractions used for fitting across the entire spectral surface, Jensen et al., 2009) and associated LCModel fits (red) are provided in Fig. 1. Prior test–retest reliability from six healthy young adults (two scans acquired one week apart) demonstrated intra-subject coefficient-of-variance of 5.3 ± 3.1% for Glu and 14.0 ± 4.8% for Gln. In the present study, average Cramer Rao lower bounds (CRLB) were 6.1 ± 2.6% for Glu and 8.9 ± 2.2% for Gln. Two adolescents and one adult only had ACC metabolite data available, and three adolescents had only POC metabolite data available.

Unsuppressed water T2 values were derived for each voxel using TE-stepped datasets and a least-squares algorithm to test for a group bias associated with using unsuppressed water as an internal reference for normalizing metabolite levels. Due to age differences in the unsuppressed water integral, Glu and Gln were normalized to creatine (Cr). Gln/Glu ratios also were calculated.

T1-weighted axial image sets were segmented into gray matter (GM), white matter (WM), and cerebrospinal fluid (CSF) binary-tissue maps (FSL, Oxford, UK). Partial tissue percentages were extracted for ACC and POC voxels (Jensen et al., 2009, Silveri et al., 2013) to quantitatively estimate potential tissue-percentage differences on metabolite ratios, which only correct for total tissue content (Choi et al., 2006).

### 2.5 Statistical analyses

Due to unequal sample sizes, FH− and FH+ groups were analyzed separately to avoid distortions of results by homogeneity of variance violations. One-way ANOVAs were used to examine age-group differences (adolescent vs. emerging adult) on Gln/Glu within the FH− and FH+ groups. An adjusted α-value of 0.025 was used to compensate for conducting two separate ANOVAs. Both gender and voxel gray-matter content (GM%) were included in initial analyses as covariates, but were non-significant contributors, and accordingly, were removed from the statistical model. Posthoc follow-up analyses were performed using one-way ANOVAs, with Gln/Cr and Glu/Cr as dependent variables, in order to determine if age-related differences in these metabolites contributed to ratio differences.

Spearman's (*ρ*) correlation analyses were conducted to examine relationships between impulsivity measures (BIS-11 subscales, Stroop derived interference time, and % errors on inhibitory trials in the Go–NoGo task) and ACC Gln/Glu for each participant group. Independent samples *t*-tests were used to examine age-group differences in impulsivity in the FH− and FH+ groups.

## 3 Results

ACC data from three subjects were identified to be statistical outliers (Gln/Glu greater than two standard deviations above the mean for the corresponding age group), and thus were removed from analyses (one FH+ adolescent and two FH− emerging adults). Significant group differences remained significant even after removal of outliers.

### 3.1 Age effects on Gln/Glu ratio in FH− and FH+

**Fig. 2:**
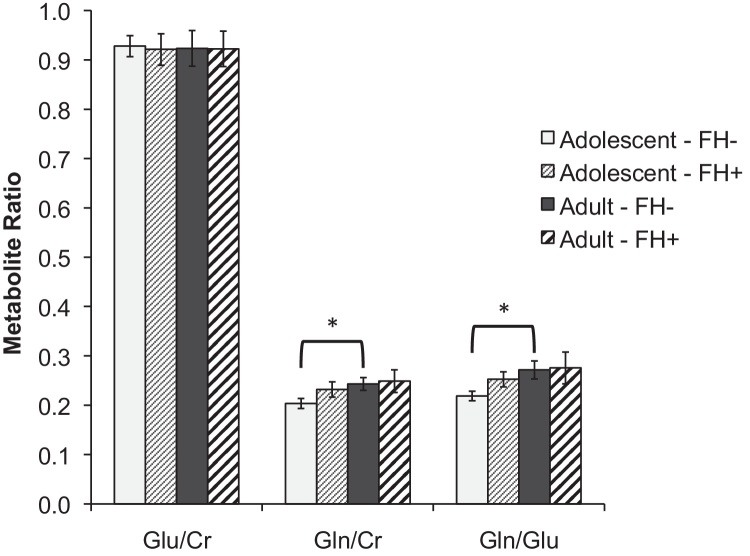
Age-related differences in Gln/Cr, Glu/Cr, and Gln/Glu ratio in FH+ and FH−. Gln/Glu and Glu/Cr ratios were significantly higher in Emerging Adults versus Adolescents in the FH− group (*p < .025).

Significantly higher %GM was observed in both regions in Emerging Adults relative to Adolescents (Table 2, ACC: *F*(1,52) = 11.64, *p* = .001, partial *η*^2^ = .183; POC: *F*(1,54) = 237.7, *p* < .001, partial *η*^2^ = .815). No significant main effects or interactions with FH status were evident. While %GM was initially included as a covariate in the analyses examining the effects of FH and age on metabolite levels, it was not a significant predictor and was therefore removed. Significantly higher ACC Gln/Glu was observed in Emerging Adults relative to Adolescents in the FH− group, *F*(1,37) = 6.44, *p* = .016, partial *η*^2^ = 0.156, but not in the FH+ group, (Fig. 2, Table 2). Follow-up analyses examining Gln/Cr and Glu/Cr differences in the FH− group revealed significantly higher Gln/Cr in Emerging Adults versus Adolescents, *F*(1,37) = 5.61, *p* = .023, partial *η*^2^ = .132 (corrected *α* = .025 due to multiple analyses), but no significant age difference in Glu/Cr (Fig. 2, Table 2). No significant age-related differences in Gln/Glu were observed in POC for the FH− or FH+ group.

**Table 2:** Metabolite concentrations in ACC and POC, values listed as mean (standard deviation).

ACC	Adolescent			Emerging adult		
	FH−	FH+	Total	FH−	FH+	Total
	*n* = 19	*n* = 8	*n* = 27	*n* = 20	*n* = 7	*n* = 27
%GM	56.4 (6.4)	59.9 (2.9)	57.4 (5.8)	62.8 (5.4)	62.8 (7.4)	62.8 (5.8)
Gln/Cr	.204 (.05)	.232 (.04)	.212 (.05)	.243 (.06)	.249 (.06)	.258 (.07)
Glu/Cr	.928 (.09)	.921 (.09)	.926 (.09)	.923 (.16)	.922 (.10)	.923 (.15)
Gln/Glu	.219 (.04)	.253 (.04)	.229 (.04)	.272 (.08)	.276 (.08)	.273 (.08)

POC	Adolescent			Emerging adult		
	FH−	FH+	Total	FH−	FH+	Total
	*n* = 18	*n* = 11	*n* = 29	*n* = 21	*n* = 6	*n* = 27
%GM	36.9 (4.7)	36.8 (4.6)	36.8 (4.6)	55.5 (4.0)	55.6 (6.4)	55.5 (4.5)
Gln/Cr	.224 (.05)	.288 (.19)	.248 (.12)	.260 (.09)	.205 (.05)	.248 (.09)
Glu/Cr	.813 (.10)	.919 (.17)	.853 (.14)	.787 (.14)	.835 (.09)	.798 (.13)
Gln/Glu	.278 (.07)	.297 (.12)	.285 (.09)	.338 (.12)	.246 (.07)	.317 (.12)

### 3.2 ACC Gln/Glu and impulsivity

**Fig. 3:**
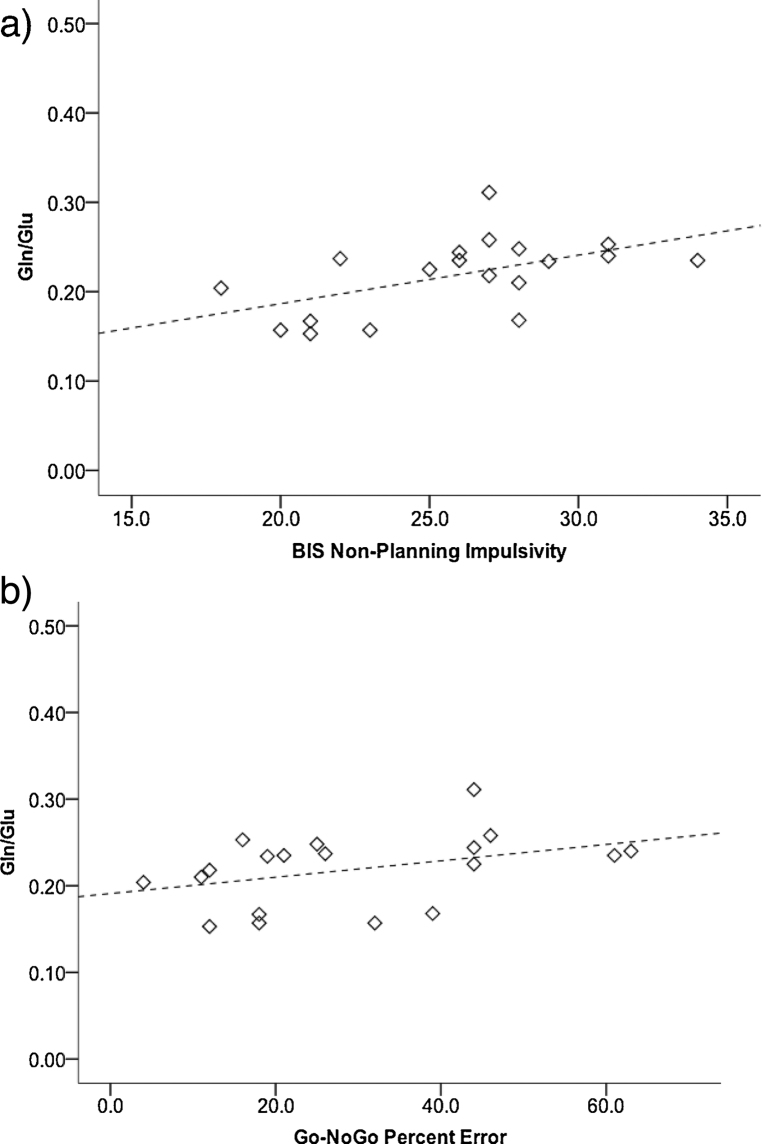
Relationships between ACC Gln/Glu ratio and impulsivity measures in FH− Adolescents. ACC Gln/Glu was significantly positively correlated with (a) BIS Non-Planning Impulsivity, *ρ* = .538, *p* = .018, and (b) higher percent error on inhibitory trials of the Go–NoGo task, *ρ* = .500, *p* = .029.

Group mean scores for BIS-11 and performance measures for Stroop and Go–NoGo tasks are provided in Table 3. In the FH− Adolescent group, a significant positive correlation was evident for Gln/Glu in ACC and Non-Planning Impulsivity on the BIS, *ρ* = .538, *p* = .018, with higher impulsivity self-report ratings being associated with higher Gln/Glu (Fig. 3a). Impulsive errors (NoGo trials) on the Go–NoGo task were also significantly positively correlated with Gln/Glu ratio in FH− Adolescents, *ρ* = .500, *p* = .029 (Fig. 3b). No significant correlations were observed between Gln/Glu and impulsivity measures in the Emerging Adult FH− group.

**Table 3:** Measures of impulsivity.

	Adolescent			Emerging adult		
	FH−	FH+	Total	FH−	FH+	Total
BIS						
Attention	15.4 (4.2)	14.4 (4.9)	15.1 (4.4)	14.1 (4.2)	14.9 (3.8)	14.3 (4.1)
Motor	24.1 (3.6)*	22.8 (4.9)	23.7 (4.0)*	19.7 (3.5)	19.1 (2.2)	19.6 (3.2)
Non-planning	25.9 (4.2)*	26.1 (6.3)	26.0 (4.8)*	20.2 (4.6)	21.2 (4.3)	20.4 (4.5)
Total	65.5 (9.1)*	63.1 (13.3)	64.8 (10.3)*	53.9 (9.6)	55.6 (8.3)	54.3 (9.1)
Stroop color naming						
Time (s)	67.2 (13.2)*	66.6 (6.1)*	67.0 (11.4)*	54.9 (8.7)	56.7 (10.3)	55.3 (9.0)
Errors	2.4 (2.0)*	2.1 (1.10)*	2.3 (1.8)*	1.0 (1.2)	1.0 (.82)	1.0 (1.1)
Stroop word reading						
Time (s)	52.1 (9.0)*	52.4 (6.1)*	52.2 (8.2)*	44.0 (7.9)	43.1 (4.6)	43.8 (7.1)
Errors	1.32 (1.2)	1.5 (1.2)	1.4 (1.1)	1.1 (1.2)	1.14 (.69)	1.1 (1.1)
Stroop interference						
Time (s)	119.1 (33.0)*	118.5 (13.6)	118.9 (28.4)*	99.7 (26.2)	92.1 (24.2)	97.7 (25.4)
Errors	4.68 (4.7)	4.1 (2.5)	4.5 (4.1)	2.6 (2.0)	3.14 (3.2)	2.7 (2.3)
Stroop der. interference						
Time (s)	51.8 (23.0)	51.9 (13.3)	51.9 (20.3)	44.8 (23.8)	35.4 (16.8)	42.4 (22.3)
Go–NoGo						
NoGo %Error	29.2 (17.3)*	29.6 (13.1)	29.3 (15.9)*	13.7 (9.2)	16.0 (14.0)	14.3 (10.4)
Go %Error	9.3 (5.3)*	10.0 (3.3)*	9.5 (4.7)*	6.2 (3.9)	3.7 (3.7)	5.5 (3.9)
Go RT	396.5 (75.3)	417.3 (41.7)	402.6 (66.9)	402.5 (67.7)	434.0 (79.4)	411.0 (70.8)

* Significant difference from corresponding emerging adult group, *p* < .05.

**Fig. 4:**
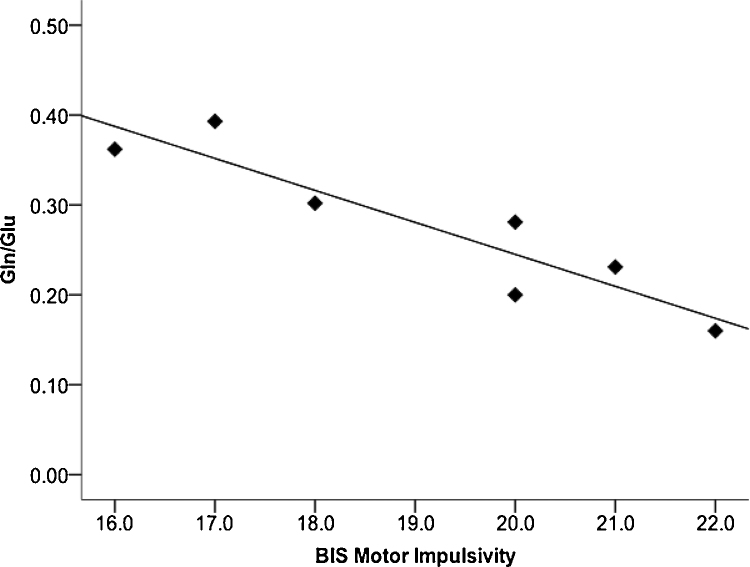
Gln/Glu ratio in ACC was negatively correlated with BIS Motor Impulsivity in the Emerging Adult FH+ group (*ρ* = −.901, *p* = .006).

While no significant correlations were found between Gln/Glu and impulsivity measures in the Adolescent FH+ group, in the Emerging Adult FH+ group, a significant negative correlation was found between ACC Gln/Glu and Motor Impulsivity on the BIS, *ρ* = −.901, *p* = .006 (Fig. 4).

## 4 Discussion

Results of the current study suggest that family history of alcoholism influences normative age-related differences in neurochemistry in the absence of heavy alcohol use. While the low-risk (FH−) adolescent group exhibited significantly lower Gln/Glu ratios than FH− emerging adult counterparts, perhaps reflecting differences in glutamatergic neurotransmission, no such age difference was observed in the high-risk (FH+) group. Inspection of mean values for each group suggests that the difference in age patterns between FH groups resulted not from lower Gln/Glu in FH+ emerging adults, but rather elevated ratios in FH+ adolescents. While high-risk adolescents (FH+) more closely resemble Gln/Glu ratios observed in emerging adults, distinct relationships were evident between Gln/Glu and impulsivity measures. A positive relationship was observed between Gln/Glu and impulsivity in FH− adolescents, which was no longer evident in emerging adults. In contrast, in FH+ participants, no significant relationships were observed in adolescents, but a strong negative relationship was observed between Gln/Glu ratios and Motor Impulsivity in emerging adults. Thus, relationships with glutamatergic neurotransmission in ACC appear to be altered in FH+ participants, following distinct developmental trajectories that vary according to FH status. It is possible that glutamatergic over-excitation elicits greater impulsivity in the healthy FH− adolescent brain but that this association is decoupled as top-down regulatory networks and GABAergic signaling become more prominent as the brain matures toward adulthood. In other words, correlates of this neurochemical signature may differ during stages of development due to reaching age-related milestones in brain maturation. The strong negative association observed between Gln/Glu in ACC and Motor Impulsivity in the FH+ emerging adults may reflect, in part, the low-risk high-risk nature of this subject group. That is, these individuals are genetically and environmentally at an elevated risk for alcohol use disorders given family history status, but have opted to not drink heavily and are light drinkers. Thus, these individuals display high levels of self-regulation, but may not rely on the same neurocircuitry to achieve this as FH− individuals. While typical adolescent brain development allows the control of impulsive actions to become increasingly automatic (Luna et al., 2010), a failure to make this developmental shift may leave FH+ adults relying heavily on recruitment of regulatory regions such as the ACC to actively self-regulate. This possibility is supported by the elevated levels of ACC activation observed during inhibitory control tasks in FH+ versus FH− individuals (DeVito et al., 2013, Hardee et al., 2014, Silveri et al., 2011). Further studies would be needed to confirm the association between glutamatergic transmission, as indexed by Gln/Glu, and activation during fMRI. Nevertheless, the findings of the current study suggest that the behavioral correlates of elevated Gln/Glu are not equivalent across age and risk groups.

Glu neurotransmission is contingent upon successful Gln-Glu cycling in the brain, perhaps increasing the combined functional relevance of these related metabolites as compared to either metabolite examined alone (Yüksel and Öngür, 2010). In this metabolic pathway, Glu that is released into the synaptic cleft following depolarization of the presynaptic membrane and is subsequently, and rapidly, taken up by astrocytes, where it is converted to Gln. Gln is then released by astrocytes, taken up by neurons, and converted back to Glu for use as a neurotransmitter. Further, in inhibitory GABAergic neurons, glutamate is converted to GABA. Thus, availability of glutamine in the brain is a critical determinant of synthesis of both glutamate in excitatory neurons and GABA in inhibitory neurons (Behar and Rothman, 2001, Rothman et al., 1999). In this regard, age-related differences in ACC GABA reported previously could also be impacted by differences in Gln level (Silveri et al., 2013).

Of particular relevance is that altered ACC Gln/Glu ratios have been associated with behavioral alterations in a number of clinical populations. For instance, reduced Gln/Glu is linked with depression and obsessive-compulsive disorder, whereas elevated Gln/Glu has been linked to mania in bipolar disorder and first psychotic break in schizophrenia (Rosenberg et al., 2005, Rosenberg et al., 2004, Yüksel and Öngür, 2010). Within borderline personality disorder and in healthy controls, Glu measures are also positively correlated with self-reported impulsivity (Hoerst et al., 2010). The impact of alcohol on glutamatergic transmission in the brain is complex. Acute alcohol exposure reduces glutamate's excitatory effects on the nervous system by disrupting the signaling cascade of NMDA receptors. Alcohol thus also impacts neural plasticity by altering levels of LTP and LDP (Gass and Olive, 2008, Krystal et al., 2003). At higher doses, ethanol can reduce extracellular glutamate in a variety of brain regions, including cingulate cortex. Chronic alcohol use, however, causes a lasting up-regulation in cortical glutamate and neural excitability (Hermann et al., 2012, Mon et al., 2012, Ward et al., 2009). FH+ individuals have been reported to display altered subjective responses to NMDA receptor antagonists, such as ketamine, relative to FH− counterparts, suggesting an important role for NMDA in the genetic vulnerability to alcoholism and substance abuse (Petrakis et al., 2004).

Interpretation of these findings is rendered more difficult by the dearth of existing developmental MRS data in healthy populations (Cohen-Gilbert et al., 2014). To date, only five studies of healthy development, all cross-sectional, report Glu and Gln metabolites, resolved using advanced spectral acquisition methods applied at high field and quantified independently, as opposed to quantifying a GLX resonance that combines these important glutamatergic components as one single peak (Gleich et al., 2014, Grachev and Apkarian, 2000, Hadel et al., 2013, Kaiser et al., 2005). Only one existing study examined developmental differences in neurometabolites between adolescence and early adulthood (Silveri et al., 2013). The current study, conducted in a larger sample size, replicates and extends our previous finding of higher Gln/Glu ratios in emerging adults relative to adolescents (Silveri et al., 2013). Importantly, taking FH status into account reveals larger effect sizes than when FH− and FH+ individuals are grouped together. Further, while there have been more than 35 MRS studies published investigating alcohol-related consequences on neurometabolites in alcohol abusing and dependent adult populations (Meyerhoff et al., 2013, Meyerhoff et al., 2014, Sullivan and Pfefferbaum, 2014), no studies published to date have examined neurochemistry using MRS in adolescents with either significant alcohol use histories or alcohol use disorders, or investigating the role of FH status (Cohen-Gilbert et al., 2014).

Notable strengths of the study include acquisition of spectral data through the use of 2D-JPRESS at 4.0T, which permits improved spatial and spectral resolution, high SNR afforded by high field MRS, and optimal detection of Glu separately from Gln. However, it should be noted that the MRS technique employed in the present study is only capable of a milimolar level of measurement, and that the observed Glu and Gln levels and ratios only reflect turnover of the general glutamatergic metabolic pool (Öngür et al., 2008, Yüksel and Öngür, 2010) While the current study included a large number of subjects for an MRS study, the current study is limited by the smaller samples sizes of FH+ individuals compared to those that were FH−. Due to the small sample sizes for FH+, findings in these groups should be considered preliminary. Accidental findings may have occurred given the multiple measures examined, however multiple comparison corrections were applied when possible. The major brain and behavior findings reported in this study are correlational in nature, and thus, follow-up investigations are necessary to better characterize neurochemical profiles and associations measures of impulsivity observed in FH− and FH+ individuals.

## 5 Conclusions

Comparing Gln/Glu and measures of inhibitory control in non-using adolescents who are FH+ or FH− for alcohol dependence may help characterize neurochemical factors that could contribute to risk for later alcohol abuse, even prior to initiation of use. The current findings suggest evidence for a distinct neurometabolite profile: elevated Gln/Glu that together with family history status may confer risk for substance use disorders in adolescents. This neurobiological vulnerability may manifest behaviorally as reduced cognitive control, which may enhance risk-taking during adolescence, but dissipates by early adulthood, possibly due to maturation of the prefrontal cortex. Identifying biomarkers of alcoholism may help target those that would benefit from early educational interventions, impulse control strategies, and delaying onset of alcohol use.

## Conflict of interest statement

The authors of this manuscript report no affiliation or involvement in any organization or entity with a financial or non-financial interest in the subject matter or materials discussed in this manuscript.

## References

[bib0005] Alterman A.I., Searles J.S., Hall J.G. (1989). Failure to find differences in drinking behavior as a function of familial risk for alcoholism: a replication. J. Abnorm. Psychol..

[bib0010] Behar K.L., Rothman D.L. (2001). In vivo nuclear magnetic resonance studies of glutamate-gamma-aminobutyric acid-glutamine cycling in rodent and human cortex: the central role of glutamine. J. Nutr..

[bib0015] Biederman J., Faraone S.V., Monuteaux M.C., Feighner J.A. (2000). Patterns of alcohol and drug use in adolescents can be predicted by parental substance use disorders. Pediatrics.

[bib0020] Casey B.J., Jones R.M., Hare T.A. (2008). The adolescent brain. Ann. N.Y. Acad. Sci..

[bib0025] Choi I.-Y., Lee S.-P., Merkle H., Shen J. (2006). In vivo detection of gray and white matter differences in GABA concentration in the human brain. Neuroimage.

[bib0030] Cohen-Gilbert J.E., Jensen J.E., Silveri M.M. (2014). Contributions of magnetic resonance spectroscopy to understanding development: potential applications in the study of adolescent alcohol use and abuse. Dev. Psychopathol..

[bib0035] Conway K.P., Swendsen J.D., Merikangas K.R. (2003). Alcohol expectancies, alcohol consumption, and problem drinking: the moderating role of family history. Addict. Behav..

[bib0040] DeVito E.E., Meda S.A., Jiantonio R., Potenza M.N., Krystal J.H., Pearlson G.D. (2013). Neural correlates of impulsivity in healthy males and females with family histories of alcoholism. Neuropsychopharmacology.

[bib0045] Finn P.R., Justus A., Mazas C., Steinmetz J.E. (1999). Working memory, executive processes and the effects of alcohol on Go/No-Go learning: testing a model of behavioral regulation and impulsivity. Psychopharmacology (Berl).

[bib0050] Fossati A., Barratt E.S., Acquarini E., Di Ceglie A. (2002). Psychometric properties of an adolescent version of the Barratt Impulsiveness Scale-11 for a sample of Italian high school students. Percept. Mot. Skills.

[bib0055] Gass J.T., Olive M.F. (2008). Glutamatergic substrates of drug addiction and alcoholism. Biochem. Pharmacol..

[bib0060] Gierski F., Hubsch B., Stefaniak N., Benzerouk F., Cuervo-Lombard C., Bera-Potelle C., Cohen R., Kahn J.P., Limosin F. (2013). Executive functions in adult offspring of alcohol-dependent probands: toward a cognitive endophenotype?. Alcohol Clin. Exp. Res..

[bib0065] Gleich T., Lorenz R.C., Pöhland L., Raufelder D., Deserno L., Beck A., Heinz A., Kühn S., Gallinat J. (2014). Frontal glutamate and reward processing in adolescence and adulthood. Brain Struct. Funct..

[bib0070] Gogtay N., Giedd J.N., Lusk L., Hayashi K.M., Greenstein D., Vaituzis A.C., Nugent T.F., Herman D.H., Clasen L.S., Toga A.W., Rapoport J.L., Thompson P.M. (2004). Dynamic mapping of human cortical development during childhood through early adulthood. Proc. Natl. Acad. Sci. U. S. A..

[bib0075] Grachev I.D., Apkarian A.V. (2000). Chemical heterogeneity of the living human brain: a proton MR spectroscopy study on the effects of sex, age, and brain region. Neuroimage.

[bib0080] Hadel S., Wirth C., Rapp M., Gallinat J., Schubert F. (2013). Effects of age and sex on the concentrations of glutamate and glutamine in the human brain. J. Magn. Reson. Imaging.

[bib0085] Hardee J.E., Weiland B.J., Nichols T.E., Welsh R.C., Soules M.E., Steinberg D.B., Zubieta J.K., Zucker R.A., Heitzeg M.M. (2014). Development of impulse control circuitry in children of alcoholics. Biol. Psychiatry.

[bib0090] Hermann D., Weber-Fahr W., Sartorius A., Hoerst M., Frischknecht U., Tunc-Skarka N., Perreau-Lenz S., Hansson A.C., Krumm B., Kiefer F., Spanagel R., Mann K., Ende G., Sommer W.H. (2012). Translational magnetic resonance spectroscopy reveals excessive central glutamate levels during alcohol withdrawal in humans and rats. Biol. Psychiatry.

[bib0095] Hill S.Y., Shen S., Lowers L., Locke J. (2000). Factors predicting the onset of adolescent drinking in families at high risk for developing alcoholism. Biol. Psychiatry.

[bib0100] Hoerst M., Weber-Fahr W., Tunc-Skarka N., Ruf M., Bohus M., Schmahl C., Ende G. (2010). Correlation of glutamate levels in the anterior cingulate cortex with self-reported impulsivity in patients with borderline personality disorder and healthy controls. Arch. Gen. Psychiatry.

[bib0105] Hu J., Yang S., Xuan Y., Jiang Q., Yang Y., Haacke E.M. (2007). Simultaneous detection of resolved glutamate, glutamine, and gamma-aminobutyric acid at 4T. J. Magn. Reson..

[bib0110] Jamadar S., DeVito E.E., Jiantonio R.E., Meda S.A., Stevens M.C., Potenza M.N., Krystal J.H., Pearlson G.D. (2012). Memantine, an NMDA receptor antagonist, differentially influences Go/No-Go performance and fMRI activity in individuals with and without a family history of alcoholism. Psychopharmacology (Berl.).

[bib0115] Jensen J.E., Licata S.C., Öngür D., Friedman S.D., Prescot A.P., Henry M.E., Renshaw P.F. (2009). Quantification of J-resolved proton spectra in two-dimensions with LCModel using GAMMA-simulated basis sets at 4 Tesla. NMR Biomed..

[bib0120] Kaiser L.G., Schuff N., Cashdollar N., Weiner M.W. (2005). Age-related glutamate and glutamine concentration changes in normal human brain: 1H MR spectroscopy study at 4T. Neurobiol. Aging.

[bib0125] Krystal J.H., Petrakis I.L., Mason G., Trevisan L., D'Souza D.C. (2003). N-methyl-D-aspartate glutamate receptors and alcoholism: reward, dependence, treatment, and vulnerability. Pharmacol. Ther..

[bib0130] Leland D.S., Arce E., Miller D.A., Paulus M.P. (2008). Anterior cingulate cortex and benefit of predictive cueing on response inhibition in stimulant dependent individuals. Biol. Psychiatry.

[bib0135] Luna B., Padmanabhan A., O’Hearn K. (2010). What has fMRI told us about the Development of Cognitive Control through Adolescence?. Brain Cogn..

[bib0140] Luna B., Sweeney J.A. (2004). The emergence of collaborative brain function: FMRI studies of the development of response inhibition. Ann. N. Y. Acad. Sci..

[bib0145] Meyerhoff D.J., Durazzo T.C., Ende G. (2013). Chronic alcohol consumption, abstinence and relapse: brain proton magnetic resonance spectroscopy studies in animals and humans. Behavioral Neurobiology of Alcohol Addiction.

[bib0150] Meyerhoff D.J., Edith V.S., Adolf P. (2014). Brain proton magnetic resonance spectroscopy of alcohol use disorders.

[bib0155] Mon A., Durazzo T.C., Meyerhoff D.J. (2012). Glutamate GABA, and other cortical metabolite concentrations during early abstinence from alcohol and their associations with neurocognitive changes. Drug Alcohol Depend..

[bib0160] Moss H.B., Talagala S.L., Kirisci L. (1997). Phosphorus-31 magnetic resonance brain spectroscopy of children at risk for a substance use disorder: preliminary results. Psychiatry Res..

[bib0165] Öngür D., Jensen J.E., Prescot A.P., Stork C., Lundy M., Cohen B.M., Renshaw P.F. (2008). Abnormal glutamatergic neurotransmission and neuronal-glial interactions in acute mania. Biol. Psychiatry.

[bib0170] Patel A.B., de Graaf R.A., Mason G.F., Rothman D.L., Shulman R.G., Behar K.L. (2005). The contribution of GABA to glutamate/glutamine cycling and energy metabolism in the rat cortex in vivo. Proc. Natl. Acad. Sci. U. S. A..

[bib0175] Petrakis I.L., Limoncelli D., Gueorguieva R., Jatlow P., Boutros N.N., Trevisan L., Gelernter J., Krystal J.H. (2004). Altered NMDA glutamate receptor antagonist response in individuals with a family vulnerability to alcoholism. Am. J. Psychiatry.

[bib0180] Poon E., Ellis D.A., Fitzgerald H.E., Zucker R.A. (2000). Intellectual, cognitive, and academic performance among sons of alcoholics, during the early school years: differences related to subtypes of familial alcoholism. Alcohol. Clin. Exp. Res..

[bib0185] Provencher S.W. (1993). Estimation of metabolite concentrations from localized in vivo proton NMR spectra. Magn. Reson. Med..

[bib0190] Rosenberg D.R., MacMaster F.P., Mirza Y., Smith J.M., Easter P.C., Banerjee S.P., Bhandari R., Boyd C., Lynch M., Rose M., Ivey J., Villafuerte R.A., Moore G.J., Renshaw P. (2005). Reduced anterior cingulate glutamate in pediatric major depression: a magnetic resonance spectroscopy study. Biol. Psychiatry.

[bib0195] Rosenberg D.R., Mirza Y., Russell A., Tang J., Smith J.M., Banerjee S.P., Bhandari R., Rose M., Ivey J., Boyd C., Moore G.J. (2004). Reduced anterior cingulate glutamatergic concentrations in childhood OCD and major depression versus healthy controls. J. Am. Acad. Child Adolesc. Psychiatry.

[bib0200] Rothman D.L., Sibson N.R., Hyder F., Shen J., Behar K.L., Shulman R.G. (1999). In vivo nuclear magnetic resonance spectroscopy studies of the relationship between the glutamate–glutamine neurotransmitter cycle and functional neuroenergetics. Philos. Trans. R. Soc. Lond. B Biol. Sci..

[bib0205] Rubia K., Smith A.B., Woolley J., Nosarti C., Heyman I., Taylor E., Brammer M. (2006). Progressive increase of frontostriatal brain activation from childhood to adulthood during event-related tasks of cognitive control. Hum. Brain Mapp..

[bib0210] Sher K.J., Martin E.D., Wood P.K., Rutledge P.C. (1997). Alcohol use disorders and neuropsychological functioning in first-year undergraduates. Exp. Clin. Psychopharmacol..

[bib0215] Sibson N.R., Dhankhar A., Mason G.F., Rothman D.L., Behar K.L., Shulman R.G. (1998). Stoichiometric coupling of brain glucose metabolism and glutamatergic neuronal activity. Proc. Natl. Acad. Sci. U. S. A..

[bib0220] Silveri M.M. (2014). GABAergic contributions to alcohol responsivity during adolescence: insights from preclinical and clinical studies. Pharmacol. Ther..

[bib0225] Silveri M.M., Rogowska J., McCaffrey A., Yurgelun-Todd D.A. (2011). Adolescents at risk for alcohol abuse demonstrate altered frontal lobe activation during Stroop performance. Alcohol. Clin. Exp. Res..

[bib0230] Silveri M.M., Sneider J.T., Crowley D.J., Covell M.J., Acharya D., Rosso I.M., Jensen J.E. (2013). Frontal lobe gamma-aminobutyric acid levels during adolescence: associations with impulsivity and response inhibition. Biol. Psychiatry.

[bib0235] Smith S., Levante T., Meier B.H., Ernst R.R. (1994). Computer simulations in magnetic resonance. An object-oriented programming approach. J. Magn. Reson. A.

[bib0240] Squeglia L.M., Jacobus J., Tapert S.F. (2009). The influence of substance use on adolescent brain development. Clin. EEG Neurosci..

[bib0245] Stoltenberg S.F., Mudd S.A., Blow F.C., Hill E.M. (1998). Evaluating measures of family history of alcoholism: density versus dichotomy. Addiction.

[bib0250] Sullivan E., Pfefferbaum A. (2014). Brain proton magnetic resonance spectroscopy of alcohol use disorders.

[bib0255] Tsai G., Coyle J.T. (1998). The role of glutamatergic neurotransmission in the pathophysiology of alcoholism. Annu. Rev. Med..

[bib0260] Ward R.J., Lallemand F., de Witte P. (2009). Biochemical and neurotransmitter changes implicated in alcohol-induced brain damage in chronic or ‘binge drinking’ alcohol abuse. Alcohol Alcohol..

[bib0265] Yüksel C., Öngür D. (2010). Magnetic resonance spectroscopy studies of glutamate-related abnormalities in mood disorders. Biol. Psychiatry.

